# Early Stage Machine Learning–Based Prediction of US County Vulnerability to the COVID-19 Pandemic: Machine Learning Approach

**DOI:** 10.2196/19446

**Published:** 2020-09-11

**Authors:** Mihir Mehta, Juxihong Julaiti, Paul Griffin, Soundar Kumara

**Affiliations:** 1 Penn State University University Park, PA United States; 2 Purdue University West Lafayette, IN United States

**Keywords:** COVID-19, coronavirus, prediction model, county-level vulnerability, machine learning, XGBoost

## Abstract

**Background:**

The rapid spread of COVID-19 means that government and health services providers have little time to plan and design effective response policies. It is therefore important to quickly provide accurate predictions of how vulnerable geographic regions such as counties are to the spread of this virus.

**Objective:**

The aim of this study is to develop county-level prediction around near future disease movement for COVID-19 occurrences using publicly available data.

**Methods:**

We estimated county-level COVID-19 occurrences for the period March 14 to 31, 2020, based on data fused from multiple publicly available sources inclusive of health statistics, demographics, and geographical features. We developed a three-stage model using XGBoost, a machine learning algorithm, to quantify the probability of COVID-19 occurrence and estimate the number of potential occurrences for unaffected counties. Finally, these results were combined to predict the county-level risk. This risk was then used as an estimated after-five-day-vulnerability of the county.

**Results:**

The model predictions showed a sensitivity over 71% and specificity over 94% for models built using data from March 14 to 31, 2020. We found that population, population density, percentage of people aged >70 years, and prevalence of comorbidities play an important role in predicting COVID-19 occurrences. We observed a positive association at the county level between urbanicity and vulnerability to COVID-19.

**Conclusions:**

The developed model can be used for identification of vulnerable counties and potential data discrepancies. Limited testing facilities and delayed results introduce significant variation in reported cases, which produces a bias in the model.

## Introduction

The continued spread of confirmed cases of COVID-19, absence of a vaccine, limited resources for testing, and assisting people with confirmed cases have presented a great challenge for our public health and health care provider systems. To this point, nonpharmaceutical interventions such as social distancing are the only effective mitigation measures. The rapid spread of the disease means that government and health services have very little time to plan and design effective response policies such as resource and workforce planning. Accurately predicting the near future COVID-19 spread at sufficient granularity would provide these organizations with better information and more time to appropriately plan and respond.

We have developed a three-stage machine learning model to estimate COVID-19 spread outcomes at the county level in the United States. In the first stage, we estimate the probability that a county has at least one confirmed COVID-19 case. In the second stage, we estimate the number of COVID-19 occurrences given a county has at least one case. Finally, we combine the results from the two stages to estimate those counties that have the greatest and least vulnerability for changes in disease prevalence for the next five-day period.

There has been significant epidemiological work for previous coronavirus pandemics such as Middle East respiratory syndrome (MERS) and severe acute respiratory syndrome (SARS) [1]. For example, Badawi et al [2] performed a systematic analysis of prevalence of comorbidities in MERS using data from 12 studies and found that diabetes and hypertension were present in 50% of the cases. Matsuyama et al [3] systematically reviewed studies involving laboratory-confirmed MERS cases to measure both the risk of admission to the intensive care unit (ICU) and death. They compared risks by age, gender, and underlying comorbidities. Park et al [4] reviewed characteristics and associated risk factors of MERS. Bauch et al [5] surveyed SARS modeling literature focused on understanding the basic epidemiology of the disease and evaluating control strategies. Surveyed SARS models varied in terms of population studied and geographical characteristics [6,7]. Different designs were used for SARS modeling, including deterministic compartmental models [7], stochastic compartmental models [6], a combination of stochastic and deterministic compartmental models [8], discrete-time models [9], logistics curve-fitting models [10], contact network models [11], and likelihood-based models [12]. Studies associated with risk factors for SARS [13] and MERS [3,14-20] have found an association between comorbidities and infected cases.

MERS and SARS epidemiological modeling has been done at different granularities such as the country [21,22], specific region [23], and case clusters [6]. Given the much broader reach of COVID-19 compared to MERS and SARS, it is very important to make predictions at a sufficiently high level of granularity. This is particularly important since previous studies have shown that there is considerable heterogeneity in space, transmissibility, and susceptibility [5]. Our approach is developed at the county level with the inclusion of a variety of health statistics, demographics, and geographical features of counties. Further, we use publicly available data so that any organization can leverage the model. To the best of our knowledge, no work has been done to predict near future infection risk at the county level using a combination of health statistics, demographics, and geographical features of counties.

## Methods

### Recruitment

We performed an epidemiological study at the US county level using publicly available data to develop a machine learning predictive model. Data analysis was performed from February 15 to April 3, 2020. The study was reviewed by the Penn State Integrated Research Ethics Board and deemed exempt because it was a deidentified, secondary data analysis. This study followed the Strengthening the Reporting of Observational Studies in Epidemiology (STROBE) reporting guideline [24].

We used US Census data to obtain county-level population statistics for age, gender, and density [25,26]. We obtained county-level data for diagnosed adult diabetics percentage and cancer crude rate statistics from the Centers for Disease Control and Prevention (CDC) [27,28]. We used county-level hypertension estimates and chronic respiratory disease mortality rates obtained from the Global Heath Data Exchange (GHDx) [29,30] website, provided by the Institute for Health Metrics and Evaluation. We obtained the centroids for each county from ArcGIS [31]. Finally, we obtained US Census Cartographic Boundary files for each county in JSON format [32] and county-level COVID-19 daily occurrences data (confirmed cases) from the NYTimes GitHub page [33,34].

### Statistical Analysis

There are three primary outcomes for our predictive model: (1) the probability that a county has at least one confirmed case of COVID-19, which we define as a positive instance; (2) the number of confirmed COVID-19 cases within a county, which we define as occurrences; and (3) vulnerability of the county.

Previous studies have shown angiotensin-converting enzyme 2 (ACE2) facilitates infection by COVID-19 [35-37], and that patients with diabetes, hypertension, and cardiovascular diseases have an increased expression of ACE2 [35]. County population factors such as density, age, and sex have a significant impact on the spread of an epidemic [38]. Cancer and chronic respiratory diseases have also been shown to increase mortality risk for COVID-19 [39]. The data set used for our three-stage model contains correlated variables. For example, diabetes and hypertension prevalence, cancer crude rate, and older adult population. Additionally, the underlying relationship between variables was assumed to be nonlinear.

### Precursor to the Prediction Model

Machine learning techniques help us to derive insights and predict trends using data without the explicit need for programming. They are mainly divided into two types based on the explicit availability of outcomes for a given set of observations: supervised and unsupervised techniques. In supervised techniques, the outcome or dependent variable is available for a given set of observations. Supervised techniques are further divided into regression or classification techniques depending upon the data type of the outcome variable: continuous or categorical [40]. In the literature, artificial neural network–based deep learning and tree-based gradient tree–boosting techniques have demonstrated better prediction capabilities in exploring nonlinear relationships among correlated predictors [41-49].

XGBoost (Extreme Gradient Boosting) [50] is a gradient tree–based supervised machine learning technique capable of performing both regression and classification tasks. The underlying algorithm combines the results from multiple individual trees with weak predictions (weak learners) to yield accurate final predictions. During the combining process, the algorithm prevents overfitting by regularizing objective function. The performance of this technique depends upon effective tuning of multiple hyperparameters such as learning rate and maximum depth with respect to underlying data distribution. These hyperparameters can be tuned with the help of random or exhaustive search as well as by using Bayesian optimization. The Bayesian optimization method has shown efficiency in terms of accuracy and time [51].

### Developing the Prediction Model

To predict COVID-19 outcomes, we divided the problem into three stages. In the first stage, we classified each county either as a positive or negative instance and used the same as a dependent variable. Hence, we built an XGBoost classifier model to learn from the data.

In the second stage, to predict number of occurrences (a continuous variable), we leveraged an XGBoost regression model that included data only for positive instances with the number of occurrences as the response.

In the last stage, we combined results from the first two stages and calculated the expected occurrences for counties as a measure of county vulnerability. For the calculation of expected occurrences, we multiplied the probability of a county belonging to the positive instances derived using the classification model, with potential occurrences the same county will have if it becomes a positive instance derived using the regression model.

### Evaluating the Prediction Model

The evaluation process is illustrated with an example for the date March 14, 2020. For this date, modeling data comprised of COVID-19 cases reported at a county level at the end of March 14 along with all other variables were obtained from fusion process.

In the first stage (classification problem), this data was divided into an 80:20 ratio for training and testing, simultaneously ensuring equivalent representation of both classes (positive and negative instance). With this setup and leveraging the HyperOpt package, multiple hyperparameters of the model were tuned using area under the receiver operating characteristic curve (AUC) and accuracy values as the evaluation criteria. The resultant model was used to compute county-level probability score.

In the second stage (regression problem), the data set was filtered to include only positive instance counties as of March 14 with number of occurrences being a dependent variable. Like the first stage, this data was divided into an 80:20 proportion for testing and training and hyperparameters were optimized by leveraging the HyperOpt package. The regression problem used the root mean squared error (RMSE) value as an evaluation criterion. The best model was used to calculate the number of occurrences associated with counties.

In the final stage, the vulnerability of a county was determined by multiplying the stage one probability score with the stage two number of occurrences. This calculated value was used to identify the riskiest and safest counties. The model is serving as a proxy for estimating after-five-day-vulnerability, the third stage outcome that was evaluated using actual COVID-19 numbers observed 5 days later, on March 19, 2020. To measure sensitivity among the top 5% riskiest counties estimated at the end of the third stage of the model, the number of counties that were observed to be positive as of March 19 were identified (Multimedia Appendix 1). The corresponding fraction was defined as sensitivity. Similarly, the specificity among the top 10% least vulnerable counties was estimated by the third stage of the model (Multimedia Appendix 2). The number of counties that continued to be observed as a negative instance were identified and the corresponding fraction was reported as specificity. The third stage model was assessed for both sensitivity and specificity.

Finally, the consistency of the three-stage modeling process was verified by repeating this process daily from March 14 to March 26 and assessing the same from March 19 to March 31.

## Results

**Figure 1:**
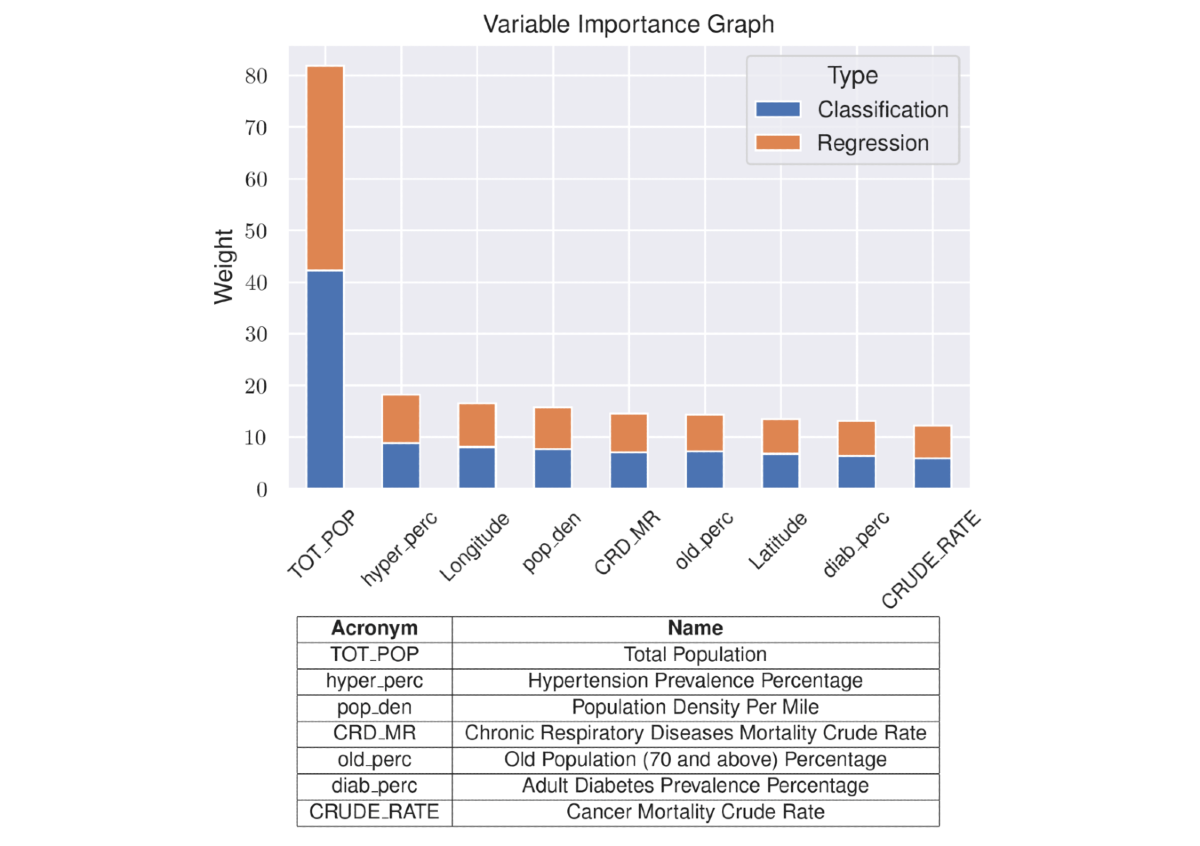
Variable importance for the classification and regression models.

The variable importance of the overlapping predictors between the final classification and regression models for March 16 is shown in Figure 1. Total population (TOT_POP) was the most important variable for both the classification and regression models. Other important variables included population density, longitude, hypertension prevalence, chronic respiratory mortality rate, cancer crude rate, and diabetes prevalence. Latitude (we use this to identify neighboring counties and the presence or absence of positive cases in the neighborhood) and the percentage of the population aged >70 years were found to be the least important features of those considered, though they still played a role.

**Figure 2:**
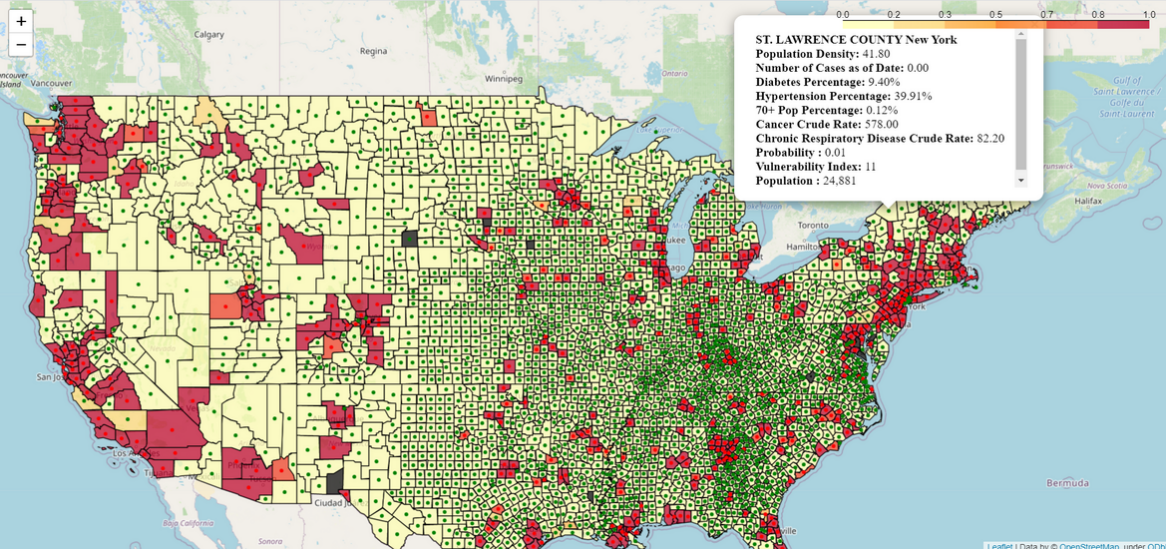
Predicted probability of there being a positive instance for each county in the United States.

Figure 2 shows a map of the United States with the predicted probability of a given county being a positive instance visualized as a color gradient. Within the software, county-level statistics can be viewed by moving the cursor over the county of interest. The example of New York County as of March 14 is shown in the Figure 2.

Accuracy and AUC for the first-stage model is shown in Table 1. Predictions of the model for all US counties are consistent over 18 days with little variation in AUC and accuracy values. Similarly, RMSE for the second-stage model for all US counties is presented in Multimedia Appendix 3. The results for first two stages of the model were evaluated until March 31.

**Table 1:** XGBoost classification training and testing details.

Data set and evaluation metrics		Mean value, %	Minimum value, %	Maximum value, %	Standard deviation, %	Number of days
**Test**						
	Accuracy	83	77	92	5	18
	Area under the curve	78	71	83	3	18
**Train**						
	Accuracy	94	82	100	5	18
	Area under the curve	91	80	100	6	18

The sensitivities and specificities for the vulnerability predictions for the three-stage model trained on data from March 14 to March 26 are shown in Tables 2 and 3. The values are given for each day. The sensitivity (Table 2) is given by the percentage of counties that had no confirmed cases but were identified as being among the 5% most vulnerable and had at least one confirmed COVID-19 case 5 days later. The specificity (Table 3) is given by the percentage of counties identified as being among the 10% least vulnerable with no confirmed cases that still had no confirmed cases 5 days later.

**Table 2:** Sensitivity of the three-stage model.

Date	Number of 5% most vulnerable counties identified on a given date (with 0 confirmed cases)	Number of counties that reported cases after 5 days	Sensitivity, %
14/3/2020	92	61	66.30
15/3/2020	119	90	75.63
16/3/2020	151	99	65.56
17/3/2020	199	144	72.36
18/3/2020	144	110	76.39
19/3/2020	176	115	65.34
20/3/2020	198	146	73.74
21/3/2020	166	125	75.30
22/3/2020	158	120	75.95
23/3/2020	84	66	78.57
24/3/2020	89	65	73.03
25/3/2020	336	208	61.90
26/3/2020	104	72	69.23

**Table 3:** Specificity of the three-stage model.

Date	Number of top 10% least vulnerable counties identified on a given date (0 confirmed cases)	Number of counties with 0 cases after 5 days	Specificity, %
14/3/2020	276	274	99.28
15/3/2020	282	276	97.87
16/3/2020	46	44	95.65
17/3/2020	313	304	97.12
18/3/2020	297	281	94.61
19/3/2020	214	198	92.52
20/3/2020	295	266	90.17
21/3/2020	312	291	93.27
22/3/2020	15	14	93.33
23/3/2020	310	289	93.23
24/3/2020	303	270	89.11
25/3/2020	214	197	92.06
26/3/2020	231	218	94.37

The data set is comprised of 37% urban and 63% rural counties based on the urban and rural county definition for 2013 [52]. To determine if there is an association between urbanicity and vulnerability, we performed a set of one-sided *t* tests. The null hypothesis that the 10% least vulnerable counties would have the same proportion of rural counties as the actual proportion of rural counties in the data set was rejected for every day from March 14 to 26. Additionally, the null hypothesis that the actual positive instances counties would have the same proportion of urban counties as the actual proportion of urban counties in the data set was also rejected for every day over the analysis period. It can therefore be concluded that there is a positive association between urban and the most vulnerable counties as well as rural and the least vulnerable counties. The continuous decreasing trend in the confidence interval of the urban counties proportion estimate within actual positive-instance counties can be used to infer that COVID-19 is propagating from urban counties to rural counties.

## Discussion

### Principal Findings

We developed a three-stage machine learning model using publicly available data to predict the 5-day vulnerability of a given US county. The model estimates the likelihood and impact that a county with no documented COVID-19 cases will have within a 5-day period and a vulnerability prediction for a county is made using those estimates. Using data from March 14 to 31, 2020, the model showed a sensitivity over 71.5% and specificity over 94%. We found a positive association between affected counties and urban counties as well as top 10% least vulnerable counties and rural counties. Further, counties with higher population density, a greater percentage of people aged >70 years, as well as higher diabetes, cardiac illness, and respiratory diseases prevalence are more vulnerable to COVID-19 than their counterparts.

Our model serves multiple purposes. First, it can help in identifying potentially vulnerable counties. This prediction would be a vital component in managing COVID-19 spread by providing vulnerability information based on the likelihood and magnitude of change within 5 days. That can help health organizations to effectively plan the management of hospital resources and the workforce, rapid response teams, COVID-19 testing kits, and COVID-19 testing locations. In addition, there are multiple counties with limited testing facilities, and with current swab-based testing, it takes multiple days to get the results. Thus, occurrences associated with each county fluctuate rapidly daily.

### Limitations

There are multiple limitations to our work. First, there are several predictors that we did not include in the model that have known associations with COVID-19. However, one of our goals was to make sure that any organization could use our model by only including data that is publicly available. Second, our analysis (Multimedia Appendix 4) found that there is an increasing trend for the coefficient of variation (CV) for occurrences associated with positive-instance counties. Note that CV is a proxy for economic inequality [53-56]. Hence, there is a bias in the response variable, which can reduce the accuracy of the prediction. As testing facilities improve in terms of numbers and efficiency, this bias would be minimized and would be reflected in the model. Given this point, it would useful to look at the riskiest and safest counties predicted by the three-stage model and examine the data for potential discrepancies. Finally, additional feature engineering and stacking methods can be used to enhance the prediction capabilities of existing models.

Our work uses open source programming and publicly available data. The full data set, sample modeling, and result outputs are available, with instructions for use [57].

### Commentary on Present Models

Presently, multiple research groups are providing COVID-19 projections on death and hospitalization case numbers. In the United States, the CDC website maintains a list of projection-providing research groups. These projections are available along with an ensemble projection. As COVID-19 approached a flattened curve stage, states deployed varied levels of easing of restrictions. Thus, these restrictions are expected to alter the presently observed dynamics of disease spread. Hence, they play an important factor in projections. To account for the same, some of these models assume stationary parameters during the projection period, while others assume some form of dynamic nature [58]. These projections are provided at different levels: country level [59], states level [60], metropolitan area level [61], and at the county level [62,63]. These projections are developed using variants of SEIR models [63], deep learning models [64], agent-based models [65], variants of mechanistic disease transmission models [66], renewal equations-based models [67], and statistical models [62]. In all these models, Columbia University’s Meta-Population SEIR Model [63] and the University of Iowa's [62] nonparametric spatial-temporal model provide projections at a county level. Columbia University’s initial model leveraged US Census county-level daily commute data during daytime and nighttime to account for the movement of the disease. However, this model does not account for county-level population heterogeneity. The University of Iowa's approach was developed using a combination of statistical and mathematical modeling techniques with an assumption of parameter-agnostic exponential family–based conditional distribution of COVID-19 cases and deaths. This model leverages county-level data on intervention policies, demographic characteristics, health care infrastructure, socioeconomic factors, urban rate, and geographical information. However, their model does not account for county-level prevalence of comorbidities. Finally, The University of Texas at Austin [61] model provides projections at the metropolitan area level using mobile-based data. With the better availability of data and information about COVID-19, current models can forecast projections for a longer period with better accuracy than our model. However, our model still presents a unique assumption-free county-level modeling approach accounting for heterogeneity using demographic, health, and geographical features.

## Appendix

Multimedia Appendix 1 Samples of counties from the top 5% riskiest counties, March 14, 2020.

Multimedia Appendix 2 Samples of counties from the top 10% safest counties, March 14, 2020.

Multimedia Appendix 3 XGBoost regression training and testing details.

Multimedia Appendix 4 COVID-19 daily positive occurrences descriptive statistics.

## Abbreviations

ACE2: angiotensin-converting enzyme 2
AUC: area under the receiver operating characteristic curve
CDC: Centers for Disease Control and Prevention
ICU: intensive care unit
MERS: Middle East respiratory syndrome
RMSE: root mean squared error
SARS: severe acute respiratory syndrome
STROBE: Strengthening the Reporting of Observational Studies in Epidemiology
XGBoost: Extreme Gradient Boosting
