# Supplementary material for: Early Stage Machine Learning–Based Prediction of US County Vulnerability to the COVID-19 Pandemic: Machine Learning Approach
Source: JMIR Public Health Surveill. 2020 Sep 11;6(3):e19446. doi: 10.2196/19446 (PMC7490002; doi:10.2196/19446)
Supplement: Multimedia Appendix 1 [file publichealth_v6i3e19446_app1.docx]

**Multimedia Appendix 1. Samples of Counties from the Top 5% Riskiest Counties- 14th March.**

| **State** | **County** | **Number of cases on March 14th** | **Number of cases on March 19^th^** |
| --- | --- | --- | --- |
| Florida | Leon | 0 | 3 |
| Illinois | Will | 0 | 11 |
| Maine | York | 0 | 3 |
| Massachusetts | Plymouth | 0 | 5 |
| Minnesota | Washington | 0 | 3 |
| New York | Erie | 0 | 27 |
| Texas | Denton | 0 | 9 |
| Wisconsin | Kenosha | 0 | 4 |

This table shows a sample list of negative instance counties as of March 14^th^. The three-stage model predicted them in the top 5% riskiest counties as of March 14^th^. All these sample counties were identified as positive instances on March 19^th^.
