# Supplementary material for: Early Stage Machine Learning–Based Prediction of US County Vulnerability to the COVID-19 Pandemic: Machine Learning Approach
Source: JMIR Public Health Surveill. 2020 Sep 11;6(3):e19446. doi: 10.2196/19446 (PMC7490002; doi:10.2196/19446)
Supplement: Multimedia Appendix 2 [file publichealth_v6i3e19446_app2.docx]

**Multimedia Appendix 2. Samples of Counties from the Top 10% Safest Counties- 14th March.**

| **State** | **County** | **Number of cases on March 14th** | **Number of cases on March 19th** |
| --- | --- | --- | --- |
| Georgia | Glascock | 0 | 0 |
| Kansas | Smith | 0 | 0 |
| Kentucky | Hickman | 0 | 0 |
| Mississippi | Issaquena | 0 | 0 |
| New Mexico | Catron | 0 | 0 |
| North Dakota | Emmons | 0 | 0 |
| Texas | Jack | 0 | 0 |
| Texas | Sutton | 0 | 0 |

This table shows a sample list of negative instance counties as of March 14^th^. The 3-stage model predicted them as the top 10% safest counties. All these sample counties continued to be negative instances on March 19^th^ as shown in the table.
