# Supplementary material for: Early Stage Machine Learning–Based Prediction of US County Vulnerability to the COVID-19 Pandemic: Machine Learning Approach
Source: JMIR Public Health Surveill. 2020 Sep 11;6(3):e19446. doi: 10.2196/19446 (PMC7490002; doi:10.2196/19446)
Supplement: Multimedia Appendix 3 [file publichealth_v6i3e19446_app3.docx]

**Multimedia Appendix 3: XGBoost Regression Training and Testing Details.**

| **Dataset** | **Evaluation Metrics** | **Mean Value** | **Minimum Value** | **Maximum Value** | **Standard Deviation** | **Number of Days** |
| --- | --- | --- | --- | --- | --- | --- |
| Test | RMSE | 117.10 | 7.13 | 372.97 | 105.00 | 18 |
| Train | RMSE | 14.02 | 1.19 | 48.59 | 14.28 | 18 |
