# Supplementary material for: Early Stage Machine Learning–Based Prediction of US County Vulnerability to the COVID-19 Pandemic: Machine Learning Approach
Source: JMIR Public Health Surveill. 2020 Sep 11;6(3):e19446. doi: 10.2196/19446 (PMC7490002; doi:10.2196/19446)
Supplement: Multimedia Appendix 4 [file publichealth_v6i3e19446_app4.docx]

**Multimedia Appendix 4: COVID-19 Daily Positive Occurrences Descriptive Statistics.**

| **Date** | **Mean** | **Standard Deviation** | **Coefficient of Variation** |
| --- | --- | --- | --- |
| 3/14/2020 | 7.01 | 25.25 | 3.60 |
| 3/15/2020 | 7.63 | 26.44 | 3.46 |
| 3/16/2020 | 8.53 | 29.39 | 3.44 |
| 3/17/2020 | 9.66 | 33.76 | 3.49 |
| 3/18/2020 | 10.96 | 38.91 | 3.55 |
| 3/19/2020 | 12.98 | 49.54 | 3.81 |
| 3/20/2020 | 14.85 | 61.98 | 4.17 |
| 3/21/2020 | 17.56 | 78.73 | 4.48 |
| 3/22/2020 | 20.93 | 103.22 | 4.93 |
| 3/23/2020 | 24.96 | 135.00 | 5.41 |
| 3/24/2020 | 28.21 | 162.05 | 5.74 |
| 3/25/2020 | 30.82 | 181.81 | 5.90 |
| 3/26/2020 | 36.29 | 218.46 | 6.02 |
| 3/27/2020 | 41.86 | 255.72 | 6.11 |
| 3/28/2020 | 47.43 | 288.14 | 6.07 |
| 3/29/2020 | 52.69 | 322.05 | 6.11 |
| 3/30/2020 | 58.01 | 354.68 | 6.11 |
| 3/31/2020 | 64.93 | 395.28 | 6.09 |
